# What is the effectiveness of radiofrequency ablation in the management of patients with spinal metastases? A systematic review and meta-analysis

**DOI:** 10.1186/s13018-021-02775-x

**Published:** 2021-11-06

**Authors:** Navanith Murali, Thomas Turmezei, Sumbal Bhatti, Puja Patel, Thomas Marshall, Toby Smith

**Affiliations:** 1 Norwich Medical School, University of East Anglia, Norwich, UK; 2 Department of Radiology, Norfolk & Norwich University Hospital, Norwich, UK

**Keywords:** Spinal metastases, Radiofrequency ablation, Spinal cord compression, Back pain, Radiotherapy

## Abstract

**Purpose:**

Spinal metastases are indicative of progressive cancer which can lead to vertebral body fractures and spinal cord compression. Radiofrequency ablation (RFA) treatment is infrequently used in patients with refractory pain. The aim of this systematic review is to determine the clinical efficacy of RFA, with the scope of using it as front-line management of spinal metastases.

**Methods:**

Electronic databases were searched (to July 2020) for studies evaluating RFA treatment for spinal metastases in adults. Measured outcomes were pain (primary), disability, health-related quality of life (HRQOL), complications, tumour control and mortality. Study inclusion, data extraction and risk of bias using the ROBIN-I tool were assessed. Meta-analysis was conducted for pooled results with homogeneity, and narrative synthesis was conducted otherwise.

**Results:**

15 studies were included. RFA reduces pain scores at 3–5 weeks [standardised mean difference (SMD 2.24, 95% confidence intervals (CI) 1.55–2.93], 3–4 months (SMD 3.00, 95% CI 1.11–4.90) and 5–6 months (SMD 3.54, 95% CI 1.96–5.11). RFA is effective in reducing disability/improving HRQOL in the short-term but longer-term efficacy remains unclear. 13.2% cases reported local tumour control failure (2.5 months–5 year follow-up) whereas mortality was 23.6% (follow-up of up to 1 year).

**Conclusion:**

Low quality evidence has proven RFA to be safe and effective in reducing pain and disability, especially in the short-term. RFA may be routinely implemented in all cases involving refractory pain or radiotherapy-resistant tumours but controlled trials are required to compare the efficacy of RFA to current frontline treatments.

*PROSPERO protocol registration number*: CRD42020202377.

**Supplementary Information:**

The online version contains supplementary material available at 10.1186/s13018-021-02775-x.

## Introduction

Bone is the most common site of metastases, affecting approximately two-thirds of cancer patients [1]. The spine is by far the most common site, making up approximately 90% of spinal masses found on imaging, with the most common primary cancers being prostate, breast, lung, kidney and thyroid tumours [2]. They clinically present with back pain and can cause metastatic spinal cord compression (MSCC) as a consequence of collapse/fracture of the affected vertebral body [3, 4]. MSCC is an oncological emergency that occurs in approximately 10% of those with spinal metastases and must be treated swiftly as it can be extremely debilitating from permanent neurological deficit [5].

In most cases, spinal metastases are a sign of incurable disease, and treatment is often palliative. According to current National Institute for Health and Care Excellence (NICE) guidelines on spinal metastases, those with painful spinal metastases without MSCC may be offered analgesia, bisphosphonates, radiotherapy, cement augmentation or surgery[6].

An alternative but not widely available treatment for spinal metastatic disease is radiofrequency ablation (RFA). RFA is an image-guided, minimally invasive procedure. It uses heat generated from current flowing through a probe (unipolar) or probes (bipolar), which upon contact with the target tumour causes coagulative necrosis and cell death [7]. Kyphoplasty and vertebroplasty are procedures often performed alongside RFA to augment the affected vertebral body and prevent further collapse [8].

At present, RFA is only used as a treatment for spinal metastases for some cases at the discretion of the spinal multidisciplinary team. The patient’s pain, prior treatments, performance status, radioresistant status of the tumour and imaging features all help determine their suitability for RFA [9].

The purpose of this systematic review is to assess the clinical efficacy (pain, quality of life, complications, mortality, tumour control) of RFA in treatment of spinal metastases with/without radiotherapy or radiotherapy alone. Although there has been some prior research on this, there is little collated evidence on long-term outcomes such as tumour recurrence and mortality [10, 11], which this study seeks to address, as well as meta-analysing the clinical outcomes.

## Methods

This systematic review was conducted in accordance with the PRISMA statement on preferred reporting items on systematic reviews and meta-analyses [12]. The protocol was registered on to PROSPERO (registration number: CRD42020202377).

### Outcomes

The primary outcomes measured were pain, disability and health-related quality of life (HRQOL). These are quantified in terms of Visual Analogue Scale (VAS) for pain and the Oswestry disability Index (ODI). The definitions of short-, mid- and long-term outcomes were subject to change once all data from included studies had been pooled.

Our secondary outcome measures were (1) tumour control/recurrence of spinal metastases; (2) mortality; and (3) complication rates.

### Information sources and search strategy

Ovid MEDLINE, EMBASE, CENTRAL databases were used to find published and unpublished studies from database inception to July 2020. Trial registries searched included WHO (World Health Organisation) clinical trial registry, clinicaltrials.gov and ISCTRN. Reference lists of included studies and similar systematic reviews were also searched. Both randomised and non-randomised study designs were part of the search criteria. Search terms were based on the PICOS framework [13]. Radiofrequency ablation OR RFA, spinal metastases OR spinal met* OR spin* adj3 met* were the main terms used. The PICOS framework used and an example search strategy can be found in Additional file 1.

### Eligibility criteria

To warrant inclusion, studies had to fulfil all the eligibility criteria; (1) both randomised and non-randomised comparator study designs; (2) study participants aged over 18-years-old; (3) study participants presenting with spinal metastases and have undergone treatment with RFA alone or RFA combined with another modality; (4) study outcomes observing our measured outcomes. Studies that only included data for primary spinal tumours, animals and RFA assisted open surgery were excluded.

### Quality assessment and Data extraction

Quality of included studies was using the Risk of Bias In Non-randomised Studies of Interventions (ROBINS-1) tool [14]. Data were extracted for: date, country of publication, trial design, participant baseline characteristics i.e. age, gender, tumour type, any additional treatments, intervention, RFA system used, co-interventions to RFA (including cement augmentation), treatment complication rates and all clinical outcomes of interest. Quality assessment and data extraction were conducted independently by two authors (NM and PP) and verified by a third reviewer (SB). An initial pilot extraction involving two studies was undertaken by all three reviewers before proceeding. Full data extraction tables can be found in Additional file 1. 

### Data synthesis analysis

In the instance where there was substantial heterogeneity between studies for study design, participant characteristics or interventions delivered, these data were analysed narratively. Where there was homogeneity, a meta-analysis was conducted for outcomes reported by two or more studies. Given the natural variability in clinical presentation and comorbid disease in people who experience spinal metastases, a random-effects model was adopted for all meta-analyses. Statistical heterogeneity was measured by the ${\mathrm{I}}^{2}$ statistic. For continuous data, meta-analyses were reported as standardised mean differences (SMD) with 95% confidence intervals (CI) and a forest plot for the primary outcome. All meta-analyses were conducted on RevMan (version 5.4.1, The Cochrane Collaboration, UK) [15].

Subgroup analysis was carried out to explore if there was substantial heterogeneity. These subgroups included patient age, type of primary cancer and RFA combination received.

Meta-analysed outcomes were assessed against the GRADE approach. Through this, each reported outcome was upgraded or downgraded by: risk of bias; imprecision; inconsistency; indirectness; and publication bias. Through this, each outcome was assessed as: very low; low; moderate or high certainty evidence.

## Results

### Study selection and characteristics

**Fig. 1:**
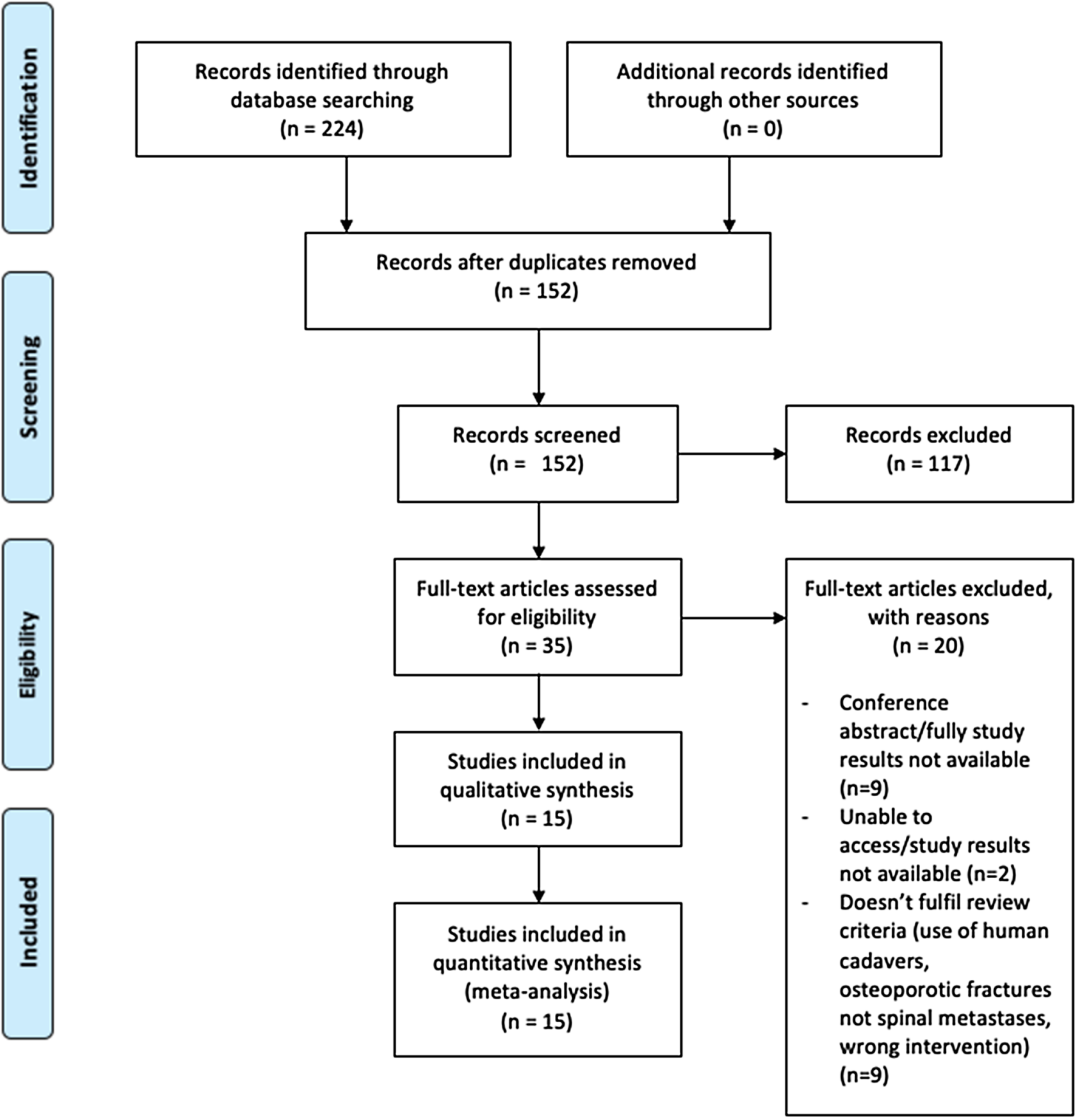
PRISMA flow diagram depicting literature search and study inclusion

The PRISMA flow diagram (Fig. 1) portrays the process of study selection [16]. Fifteen studies were included for this systematic review [17–31].

Study characteristics are summarised in Table 1. The 15 eligible studies involved a total of 725 patients. Only two studies were comparative in nature; Prezzano et al. compared outcomes for RFA and cement augmentation versus RFA and radiotherapy[19] whereas Proschek et al. compared RFA by itself to RFA with cement augmentation [28]. All other studies observed outcomes for one group all receiving the same RFA intervention. Fourteen studies measured pain outcomes. Five studies measured disability/quality of life outcomes. Ten studies also observed tumour control as an outcome, and 10 studies also included mortality statistics. The most common primary tumours for patients in the studies included were breast, renal and lung neoplasms. All studies included were non-randomised.

**Table 1:** Summary of characteristics of included studies

Study (study design)	Country	Study outcomes	Ablation system used ± combined intervention used	Comparator used (if available)	No. of patients enrolled to study (male/ female)	Mean age of patients (SD)	Most common primary tumour (*n)*	Time points for assessment of outcomes
Bagla et al. [17] (multicentre prospective clinical series)	USA	Pain, disability, complications	STAR tumour ablation system + vertebral augmentation	–	50 (26/24)	61 (13)	Renal (11)	Baseline, prior to discharge, 3 days, 1 week, 1 month and 3 months
Sayed et al. [18] (single centre prospective cohort study)	USA	Pain, quality of life and local tumour control	STAR tumour ablation system + vertebral augmentation	–	30 (19/11)	62.9 (13.45)	Renal (7)	Baseline, 3 days, 1 week, 1 month and 3 months
Prezzano et al. [19] (single centre retrospective study)	USA	Pain and local tumour control	OsteoCool ablation device + vertebral augmentation	RFA + radiotherapy (3D-CRT, volumetric-arc and SBRT techniques)	26	63	Lung, breast (8)	Baseline, and median of 3 and 12 weeks for pain. Tumour progression was assessed as when it re-occurred- medial follow up was 8.2 months
Tomasian et al. [20] (single-centre retrospective study)	USA	Local tumour control and complications	STAR tumour ablation system + vertebral augmentation	–	27 (17/10)	–	Lung (10)	2 h, 1 day, 1 week and 1 month for complications. Medial follow up of 16 weeks for tumour control
Zhao et al. [21] (single centre prospective study)	China	Pain, quality of life and complications	RFA-I type multipolar cancer ablation system + vertebral augmentation	–	16 (4/12)	66.8	Lung (9)	Tumour control within 1 week Pain at baseline, 24 h, 48 h, 72 h, 1 week, 1 month, 2 months, 3 months and 6 months QoL at baseline and 1 month
Cazzato et al. [22] (single centre retrospective study)	France	Pain, complications and local tumour control	OsteoCool ablation system + vertebral augmentation	–	11 (5/6)	61.3 (11.6)	Lung (4)	Baseline, 1–2 months for pain, routine imaging for tumour control
Greenwood et al. [23] (single centre retrospective study)	USA	Pain and local tumour control	STAR tumour ablation system and vertebral augmentation + radiotherapy	–	21 (13/9)	61.8	Lung (8)	Baseline, 1 week, 1, 3 and 6 months
Anchala et al. [24] (multicentre retrospective review)	USA	Pain, local tumour control, complications	STAR tumour ablation system (+ vertebral augmentation if required)	–	92 (13/21^a^)	60^a^	Lung	Baseline, 1 week, 1 month and 6 months
Gervagez et al. [25] (single-centre retrospective study)	Germany	Pain, disability and tumour control	Radionics system or RITA system + vertebral augmentation	–	41 (25/16)	62.7 (9)	Breast (8)	Baseline, 6 weeks, 6 months and more than 6 months
Wallace et al. [26] (single-centre retrospective study)	USA	Pain and complications	STAR tumour ablation system + vertebral augmentation	–	72 (28/44)	68.4 (18.8)	Lung (20)	Baseline, 1 and 4 weeks
Zheng et al. [27] (single-centre retrospective study)	China	Pain, tumour control, complications	RFA (unknown system) + vertebral augmentation	–	26 (12/14)	59.31 (11.62)	Breast (6)	Baseline, 3 days, 1 week, 1 month, 3 months and 6 months Mean follow up of 8.4 months for tumour control
Proschek et al. [28] (prospective pilot study)	Germany	Pain, quality of life, complications, tumour control	CelonPOWER system	RFA + cement augmentation	16 (0/16)	59.5	Breast (16)	Mean follow up of 20.4 months (Range 8–36)
Dabravolski et al. [29] (single centre retrospective study)	Germany	Pain, tumour control and survival	CAVITY SpineWand + vertebral augmentation	–	250 (94/156)	65.6	Breast (48)	Baseline, 2 and 14 days, 3, 12, 24, 36, 48 and 60 months
Georgy et al. [30] (single centre retrospective study)	USA	Pattern of cement deposition, pain, complications	CAVITY SpineWand + vertebral augmentation	–	37 (16/21)	69.6	Breast (10)	Baseline, 2–4 weeks after
Nakatsuka et al. [31] (single centre prospective study)	Japan	Pain, efficacy, complications	Cool-tip RF ablation system (+ vertebral augmentation if required)	–	10 (6/4)	61.0 (13)	Colorectal (4)	Mean follow up of 4.5 months (range 2.7–7.1)

^a^Data from largest institution only

Summary of risk of bias assessment is summarised in Table 2. All studies were assessed as serious risk for bias overall mainly due to confounding factors, subjectivity in measurement of outcomes and high dropout rate. The overall strength of evidence using the GRADE approach ranged from ‘low’ to ‘very low’ (Additional file 1).

**Table 2:** Quality assessment of included studies using ROBINS-1 tool

Study	Bias due to confounding	Bias in selection of participants into study	Bias in classification of interventions	Bias due to deviations from intended interventions	Bias due to missing data	Bias in measurement of outcomes	Bias in selection of the reported result	Overall risk of bias
Bagla et al. [17]	Serious	Low	Low	Low	Moderate	Serious	Low	Serious
Sayed et al. [18]	Serious	Low	Low	Low	Moderate	Serious	Low	Serious
Prezzano et al. [19]	Serious	Low	Low	Low	Low	Serious	Low	Serious
Tomasian et al. [20]	Serious	Low	Low	Low	Moderate	Low	Low	Serious
Zhao et al. [21]	Serious	Low	Low	Low	Low	Serious	Low	Serious
Cazzato et al. [22]	Serious	Moderate	Low	Low	Low	Serious	Low	Serious
Greenwood et al. [23]	Serious	Low	Low	Low	Low	Serious	Low	Serious
Anchala et al. [24]	Serious	Low	Low	Low	Moderate	Serious	Low	Serious
Gervagez et al. [25]	Serious	Low	Low	Low	Serious	Serious	Low	Serious
Wallace et al. [26]	Serious	Low	Low	Low	Low	Serious	Low	Serious
Zheng et al. [27]	Serious	Low	Low	Low	Low	Serious	Low	Serious
Proschek et al. [28]	Moderate	Low	Low	Low	Low	Serious	Low	Serious
Dabravolski et al. [29]	Serious	Low	Low	Low	Low	Serious	Low	Serious
Georgy et al. [30]	Serious	Low	Low	Low	Moderate	Serious	Low	Serious
Nakatsuka et al. [31]	Serious	Low	Low	Low	Low	Serious	Low	Serious

All studies are found to have serious risk of bias overall which is particularly attributed to bias due to confounding and measurement of outcomes

### Pain

Altogether, nine studies included pain data which could be statistically pooled for meta-analyses [18, 19, 21–24, 26, 27, 31]. Though there was high methodological heterogeneity due to the serious risk of bias in non-randomised studies, the clinical diversity among these studies was quite similar as participants had similar ages, similar RFA systems being used and similar primary tumours. We could not conduct any sub-group analyses as the majority of these studies only included pooled data and no individual pain scores. Time points for short-, mid- and long-term pain were adjusted to 3–5 weeks, 3–4 and 5–6 months, respectively, to allow for pooling (Additional file 1).

### Effect of radiofrequency ablation on short-term pain

Eight studies included pain scores at 3–5 weeks follow-up which could be statistically pooled [18, 19, 21–24, 26, 27]. Evidence of low quality showed that RFA improves pain short-term as portrayed by the reduction in VAS score at 3–5 weeks difference (SMD 2.24, 95% CI 1.55–2.93, ${\mathrm{I}}^{2}=89\%$, eight studies, 286 participants).

### Effect of radiofrequency ablation on mid-term pain:

Four studies included pain data at 3–4 months follow-up which could be pooled [18, 19, 21, 27]. Evidence of very low strength showed that RFA also improves pain mid-term (SMD 3.00, 95% CI 1.11–4.90, ${\mathrm{I}}^{2}=95\%$, four studies, 98 participants). Though there is a clear improvement in pain at this time-point, the extent to which it is pain-effective is unclear due to wide confidence intervals.

### Effect of radiofrequency ablation on long-term pain:

Four studies included pain data at 5–6 months follow-up [21, 23, 26, 31]. Evidence of very low strength showed that RFA does improve pain with reduced VAS scores longer-term (SMD 3.54, 95% CI 1.96–5.11, ${\mathrm{I}}^{2}$=88%, four studies, 144 participants). Similar to the mid-term outcome, the confidence interval is wide but still shows an overall significant reduction in pain 5–6 months following RFA.

### Disability and Health-Related Quality of Life

Five studies measured disability or HRQOL outcomes, using a variety of scales and indexes [17, 18, 21, 25, 28]. A meta-analysis was not conducted for this outcome as only one study reported variance data and could be statistically pooled.

### Short-term effect of RFA on disability and HRQOL (< 3 months)

Four out of five studies reported a significant reduction in disability and/or improvement in HRQOL at less than three months of follow-up following RFA treatment. Bagla et al. reported a significant decrease in ODI score (i.e. reduction in disability) by 7.7% (*P* < 0.01) at Day 3 of follow up and 12.9% decrease at one month follow up (*P* < 0.01) [17]. Similarly, Proschek et al. also reported a significant 30% decrease in ODI for both the RFA only arm (*P* < 0.014) and RFA plus vertebral augmentation arm (*P* < 0.0031) following RFA [28]. Gervagez et al. also reported a significant reduction in disability following RFA. This was measured as an 8% decrease in pain disability index score (PDI) at six weeks (*P* < 0.015) [25].

Bagla also reported mean increases in FACT-G7 and FACT-BP scores (i.e. improvement in quality of life), with an increase of 4.8 (*P* < 0.0001) and 14.7 (*P* < 0.0001) at one month, respectively [17]. Sayed et al. also measured FACT-G7 scores but did not observe a significant difference [18]. Zhao et al. reported significant improvements in physical function (*P* = 0.03) and emotion function (*P* = 0.003) using the EORTC QLQ-C30 scale at one month follow-up [21].

### Mid-term effect of RFA on disability and HRQOL (3–12 months)

Four studies measured disability and/or HRQOL outcomes at 3–12 months. At three months, Bagla et al. and Gervagez et al. reported a 15.9% decrease in ODI (*P* < 0.01), and a 4% decrease in PDI [0.002] scores, respectively [17, 25]. Sayed et al. did not observe a significant improvement in FACT-G7 score at three months post-RFA. (*P* = 0.071), whereas Proschek et al. did not find a significant reduction in mean ODI score at 3–6 months follow-up (*P* = 0.06) [18, 28].

### Long-term effect of RFA on disability and HRQOL (> 12 months)

Only Proschek et al. reported disability and HRQOL outcomes past 12 months [28]. It concluded that there was not a significant decrease in ODI scores at follow up of 15–36 months (*P* = 0.071). Gervagez et al. also reported a 10% decrease in PDI at over six months of follow up (*P* = 0.003) [25]. Since the exact follow up period was not stated, we cannot determine whether this decrease would fall under mid- or long-term effect RFA.

### Complications

All studies except one included complication data [19]. Cement extravasation was by far the most common complication, occurring in 10.3% of cases. Out of these seventy-two occurrences, only one was deemed clinically significant, causing moderate pain and requiring surgical removal [21]. Other complications reported were radicular pain, paraplegia and transient neural damage, all of which were temporary. Cazzato et al. reported the only case of sepsis, which is a major complication and resulted in death of the patient [22]. In this case, the patient received RFA despite having a subclinical paravertebral abscess which was misdiagnosed. Excluding this human error, radiofrequency ablation for the treatment of spinal metastases has otherwise proven to be safe.

### Tumour control

Ten studies reported data on tumour control and recurrence [18–20, 22–25, 27–29]. In total, 51 out of 387 patients (13.2%) showed failure of tumour control or demonstrated tumour recurrence (2.5 month to five years follow up). It must be noted that the differences in tumour histology and follow-up periods between the studies will likely have affected this result.

### Mortality

Ten studies included mortality data [17, 19–23, 26, 27, 29, 31]. Out of 462 participants, 109 died (23.6%) at a median/mean follow of up to one year.

Bagla et al. and Tomasian et al. both attributed the deaths of the 13 patients in their studies to causes unrelated to procedure[17–20], and post-operative sepsis was the cause of the single death in Cazzato et al. [22]. Cause of death was not mentioned in the rest of the studies. Dabravolski et al. [29] provided survival data for up to five years; 34.1% of patients died within one year, 58.9% of patients died within two years, 76.9% at three years, 83.8% at four years and 85.1% at five years.

Due to the ambiguity in the cause of deaths and the already reduced life expectancy of those with metastatic cancer, it is difficult to form a strong association between RFA and survival.

### RFA alone versus RFA and radiotherapy

Two studies observed RFA combined with radiotherapy (RT) specifically as a combined multimodality treatment [19–23]. Prezzano et al. determined a case as RFA plus RT if patients had received RT to the same spinal level they had received RFA without any local progression. Greenwood et al. determined a case as RFA plus RT if they received either of the treatments to the same level within four weeks of each other. Both studies observed a significant pain reduction in such circumstances. Prezzano et al. also included a control group which received RFA by itself and found there was no significant difference in VAS scores between the RFA group versus the RFA + RT group (*P* = 0.96). Though there is some evidence that RFA plus RT is effective in pain relief, it is still unclear whether it is more effective as a combination treatment compared to RFA alone.

Prezzano et al. also measured tumour control and survival data for both RFA and RFA plus RT groups [19]. The RFA plus RT group showed better local tumour control (1/11 patients had local failure in RFA + RT group versus 8/17 in RFA only group). Median survival was also longer in the combination group (55.3 weeks vs 31.9 weeks in RFA only group).

## Discussion

The primary objective of this systematic review was to assess the clinical efficacy of RFA in patients with spinal metastases. We report that radiofrequency ablation, whether by itself or with combination treatment, has some evidence that it is effective in reducing pain and disability in patients with spinal metastases, especially in the short-term. The extent to which RFA reduces pain is unclear in the mid- and long-term due to the wide confidence intervals in the meta-analyses. This is similar for disability and HRQOL as results are unclear at longer time points. Since patients with spinal metastases are at an advanced stage of cancer, it is understandable for disability and HRQOL outcomes to worsen with mid- and long-term outcomes due to progression of other cancer-related sequelae. Except for one case of sepsis which was attributed to human error, there were no major complications attributed to the RFA procedure itself, and therefore, all studies deemed it to be safe.

The two previous systematic reviews conducted on this subject found that RFA may be safe and effective as analgesia in the short-term (one week to six months) [10, 11]. The results of this review and meta-analysis support and further this by analysing deeper into outcomes such as disability, tumour control and mortality while also finding minimal evidence for RFA in combination with RT.

We are unable to draw any clear conclusions on the effect of RFA on local tumour control and mortality. The difference in follow-up period between studies is likely to obscure the true effect of RFA on tumour control as participants in longer follow-up studies might be at increased risk of local failure due to the progressive nature of metastatic cancer. This was also the case for mortality data. Additionally, many of the studies that reported mortality data did not mention cause of death, so we could only group this data as all-cause mortality.

Our second objective was to assess the efficacy of RFA as combined treatment with another intervention. Though there was evidence to suggest RFA is more effective combined with radiotherapy, it remains unclear if RFA with radiotherapy is more effective than RFA alone for reducing pain.

### Limitations

The main limitation of this review was the statistical heterogeneity presented by the meta-analyses. This could be attributed to the methodological heterogeneity due to the serious risk of bias in all studies. Moreover, we were unable to conduct sub-group analyses to explore heterogeneity as most studies only included pooled data. Though authors were contacted for individual participant data, no response was received. As a result of this, the evidence in this review is of low quality. We also found no RCTs, so all studies included were non-randomised. Their absence in the context of spinal metastases is understandable as patients have a poor prognosis, which would likely impact on RCT recruitment and completion. As a result, confounders and lack of blinding in the included studies meant that all were assessed as serious risk of bias. One such confounder was adjuvant oncological treatments received by patients (e.g. chemotherapy) which could have affected spinal metastases outcomes irrespective of RFA. Some studies mentioned if patients received these treatments, but it is hard to exclude their effect without RCT design in place. Most studies also did not include a comparator group, and therefore, there was no way of knowing whether RFA improved or worsened these outcomes such as mortality unless they were compared to participants who received no RFA or a different spinal metastases treatment e.g. radiotherapy. Another limitation was the absence of a funnel plot for assessment of publication bias. This was omitted as it is not recommended when there are less than ten studies in the meta-analysis, as the power of the tests is too low to distinguish from real asymmetry [15].

### Implications for clinical practice and future research

Currently, RFA is not part of NICE guidelines in the management of spinal metastases and is only used infrequently in the UK [6]. This systematic review has found evidence that RFA is a safe procedure which is effective in pain and disability reduction, especially in the short-term. There is also some weak evidence on the benefits of RFA being combined with radiotherapy. Though there was a variety of tumour histology in each study, we believe the collated study population represented typical oncological patients who may present with spinal metastases, since many different primary tumours can metastases to the spine. With these results in mind, RFA could potentially be implemented as a treatment for refractory pain following conventional analgesia and radiotherapy, usually followed by vertebral cement augmentation as this was common practice across all included studies.

Randomised controlled trials are needed to definitively assess the efficacy of RFA compared to standard treatments such as radiotherapy by providing higher quality evidence on the true effectiveness of RFA on clinical outcomes, especially tumour control and mortality (e.g. survival analyses and including cause of death). This would provide higher quality evidence on how RFA could be used alongside or even ahead of radiotherapy. Such trials could also include participants with a specific primary tumour only, e.g. targeting spinal metastases in a typically radioresistant tumour such as renal cell carcinoma. Without such trials, it is difficult to assess the role of RFA treatment ahead of current standard treatments such as radiotherapy.

## Conclusion

We report evidence of low-quality suggesting radiofrequency ablation (RFA) is safe and effective in reducing pain and disability, as well as improving quality of life in patients with spinal metastases in the short-term especially. The results of this review may justify the use of RFA in refractory cases, in particular radioresistant tumours. There is limited evidence comparing RFA to radiotherapy, and thus, we are unable to draw conclusions on tumour control and mortality without conducting higher-quality studies such as randomise control trials.

## Supplementary Information

**Additional file 1.** From top to bottom- **Table S1.** PICOS framework for the search strategy. **Figure S1.** The random-effects meta-analysis showing the effect of RFA on pain at 3-5 weeks, 3-4, and 5-6 months. There is significant pain reduction at all these time points. **Table S2.** Summary of complications reported across all included studies. **Table S3.** Summary of reported tumour control and mortality data across all included studies. **Table S4.** Example search strategy used for the CENTRAL database. **Table S5.** Full data extraction table for patient baseline characteristics. **Table S6.** Summary of GRADE approach for meta-analysed outcomes. **Table S7.** Data extraction table for pain, disability and HRQOL. **Table S8.** Full data extraction table for complications, mortality and tumour control.

## Data Availability

All data generated or analysed during this study are included in this published article (and its Additional file 1).

## Abbreviations

RFA: Radiofrequency Ablation
HRQOL: Health-related Quality of Life
RT: Radiotherapy
CI: Confidence interval
SMD: Standardised mean difference
NICE: National Institute for Health and Care Excellence
MSCC: Metastatic Spinal Cord Compression
VAS: Visual Analogue Scale
ODI: Oswestry Disability Index
ROBINS-1: Risk of Bias In Non-randomised Studies of Interventions
WHO: World Health Organisation
