# Supplementary material for: What is the effectiveness of radiofrequency ablation in the management of patients with spinal metastases? A systematic review and meta-analysis
Source: J Orthop Surg Res. 2021 Nov 6;16:659. doi: 10.1186/s13018-021-02775-x (PMC8571892; doi:10.1186/s13018-021-02775-x)
Supplement: Supplementary file 1 — Additional file 1. From top to bottom- Table S1. PICOS framework for the search strategy. Figure S1. The random-effects meta-analysis showing the effect of RFA on pain at 3-5 weeks, 3-4, and 5-6 months. There is significant pain reduction at all these time points. Table S2. Summary of complications reported across all included studies. Table S3. Summary of reported tumour control and mortality data across all included studies. Table S4. Example search strategy used for the CENTRAL database. Table S5. Full data extraction table for patient baseline characteristics. Table S6. Summary of GRADE approach for meta-analysed outcomes. Table S7. Data extraction table for pain, disability and HRQOL. Table S8. Full data extraction table for complications, mortality and tumour control. [file 13018_2021_2775_MOESM1_ESM.docx]

**Supplementary information**

**Table S1.PICOS framework for the search strategy:**

| **Population** | Patients with confirmed diagnosis spinal metastases |
| --- | --- |
| **Intervention** | Radiofrequency ablation |
| **Comparison** | Nothing, RFA combined with another modality e.g. radiotherapy + RFA |
| **Outcome** | Pain, disability and quality of life, mortality, tumour control, |
| **Study design** | Randomised and non-randomised quantitative studies |

**Figure S1. Effect of RFA on pain at 3-5 weeks, 3-4 and 5-6 months using a random-effects meta-analysis**

***
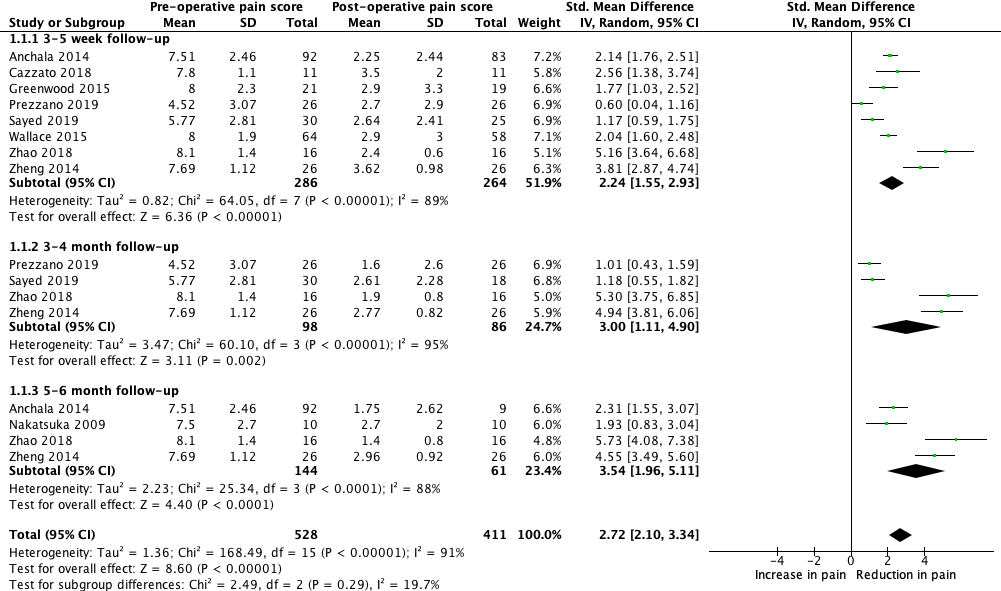
***

**Table S2. Data from studies which reported procedural and patient-reported complications:**

| **Complication** | **Number studies reported (and *n*)** | **Number of events** | **% of cases** |
| --- | --- | --- | --- |
| Cement extravasation | 14 (699) | 72 | 10.3% |
| Radicular pain | 14 (699) | 9 | 1.3% |
| Paraplegia | 14 (699) | 1 | 0.1% |
| Transient neural damage | 14 (699) | 1 | 0.1% |
| Sepsis | 14 (699) | 1 | 0.1% |

**Table S3. Summary of reported tumour control/recurrence and mortality data:**

|  | **Follow up period** | **Number studies reported (and *n*)** | **Number of events** | **% of cases** |
| --- | --- | --- | --- | --- |
| **Local tumour recurrence/progression** | 2.5 month – 5 years follow up | 10 (387) | 51 | 13.2% |
| **All-cause mortality** | median follow up of up to 1 year | 9 (462) * | 109 | 23.6% |

**Prezzano et al also mentioned median survival was 31.9 weeks in RFA alone vs 55.3 weeks in RFA +RT but no exact figure about number of people surviving was mentioned hence why it wasn’t included in the table.*

**Table S4. Example of search strategy that was used for CENTRAL:**

|  | **Search terms** | **Search results** |
| --- | --- | --- |
| #1 | MeSH term [Ablation technique] explode all trees | 5718 |
| #2 | Radiofrequency ablation | 3104 |
| #3 | RFA | 1115 |
| #4 | #1 OR #2 OR #3 | 8184 |
| #5 | Spin* adj3 met* | 460 |
| #6 | Spinal met* | 19055 |
| #7 | #5 OR #6 | 19200 |
| #8 | #7 AND #4 | 84 |

**Table S5. Data extraction table for patient baseline characteristics:**

| Study | Mean age of patients (SD) | Gender of patients (male/female) | Primary tumour (*n)* | | No. of vertebral lesions | Location of lesions *(n)* | Any prior additional treatment received *(n)* |
| --- | --- | --- | --- | --- | --- | --- | --- |
| Bagla et al 2016 | 61 (13), range 23-83 | 26/24 | Renal | 11 | 69 | Thoracic (30), lumbar (39) | Radiation therapy (16) |
|  |  |  | Breast | 10 |  |  |  |
|  |  |  | Lung | 9 |  |  |  |
|  |  |  | Liver | 3 |  |  |  |
|  |  |  | Bladder | 2 |  |  |  |
|  |  |  | Adenocarcinoma, bile duct, cervical, colon, head and neck, maxillary sinus, melanoma, ovarian, pancreatic, prostate, thyroid, uterine | 1 |  |  |  |
|  |  |  | Cancer not specified | 3 |  |  |  |
| Sayed et al 2019 | 62.9 (13.45) | 19/11 | Renal | 7 | 34 | Thoracic (13), lumbar (21) | - |
|  |  |  | Breast | 6 |  |  |  |
|  |  |  | Lung | 5 |  |  |  |
|  |  |  | Liver , bladder, melanoma | 2 |  |  |  |
|  |  |  | Adenocarcinoma, multiple myeloma, colon, maxillary sinus, prostate, thyroid | 1 |  |  |  |
| Prezzano et al 2019 | 63.0, range 32-75 | - | Lung, breast | 8 | 28 | All tumours were thoracic or lumbar | Radiation therapy (10) |
|  |  |  | Oesophagus | 3 |  |  |  |
|  |  |  | Colon, liver, bone | 2 |  |  |  |
|  |  |  | Renal, pancreas, parotid | 1 |  |  |  |
| Tomasian et al 2018 | Range 23-86 | 17/10 | Lung | 10 | 33 | Thoracic (12), lumbar (20), sacral (1) | Radiation therapy (7) |
|  |  |  | Sarcoma | 6 |  |  |  |
|  |  |  | Renal | 4 |  |  |  |
|  |  |  | Melanoma | 3 |  |  |  |
|  |  |  | Epithelioid haemangio-endothelioma, multiple myeloma | 2 |  |  |  |
|  |  |  | liver, head and neck, breast, bladder, prostate, germ cell | 1 |  |  |  |
| Zhao et al 2018 | 66.8, range 54-84 | 4/12 | Lung | 9 | 30 | Thoracic (19), lumbar (9), sacral (2) | - |
|  |  |  | Liver | 3 |  |  |  |
|  |  |  | Breast | 2 |  |  |  |
|  |  |  | Rectal, lymphoma | 1 |  |  |  |
| Cazzato et al 2018 | 61.3 (11.6), range 41-73 | 5/6 | Lung | 4 | 11 | Thoracic (2), lumbar (8), sacral (1) | Radiotherapy (3), arterial embolization (1) |
|  |  |  | Liver | 2 |  |  |  |
|  |  |  | Breast | 2 |  |  |  |
|  |  |  | Bladder, chondrosarcoma, colorectal | 1 |  |  |  |
| Greenwood et al 2015 | 61.8, range 30-84 | 13/9 | Lung | 8 | 36 | Thoracic (15), lumbar (21) | Radiotherapy (22) |
|  |  |  | Renal | 5 |  |  |  |
|  |  |  | breast | 3 |  |  |  |
|  |  |  | Colorectal | 2 |  |  |  |
|  |  |  | Bladder, angiosarcoma, melanoma | 1 |  |  |  |
| Anchala et al 2014 | 60, range 35-84* | 13/21* | Lung | (27%) | 128 | Thoracic (50%), lumbar (39%), sacral (11%)* | Radiotherapy (17), chemotherapy (24) * |
|  |  |  | Breast | (16%) |  |  |  |
|  |  |  | Sarcoma | (9%) |  |  |  |
| Gervagez et al 2008 | 62.7(9), range 46-82 | 25/16 | Breast | 8 | - | - | All patients had tumours resistant to previous chemotherapy, radiotherapy, hormone therapy and surgery |
|  |  |  | Multiple myeloma | 6 |  |  |  |
|  |  |  | GI tract, prostate | 5 |  |  |  |
|  |  |  | Renal, thyroid | 4 |  |  |  |
|  |  |  | Melanoma | 3 |  |  |  |
|  |  |  | Cervical | 2 |  |  |  |
|  |  |  | Pancreatic, bladder, osteosarcoma, leiomyoma | 1 |  |  |  |
| Wallace et al 2015 | 68.4 (18.8) | 28/44 | Lung | 20 | 110 | Thoracic (54), lumbar (56) | Radiotherapy (22) |
|  |  |  | Sarcoma | 13 |  |  |  |
|  |  |  | Breast | 11 |  |  |  |
|  |  |  | Renal | 9 |  |  |  |
|  |  |  | GI tract, multiple myeloma, melanoma | 4 |  |  |  |
|  |  |  | Other | 7 |  |  |  |
| Zheng et al 2014 | 59.31 (11.62), range 32-75 years | 12/14 | Breast | 6 | 38 | Thoracic (11), lumbar (24), sacral (3) | - |
|  |  |  | Prostate | 5 |  |  |  |
|  |  |  | Lung | 3 |  |  |  |
|  |  |  | Renal, liver, sacrum, lymphoma | 2 |  |  |  |
|  |  |  | Mesenchymal, oesophagus, adenocarcinoma, thyroid | 1 |  |  |  |
| Proschek et al 2009 | 59.5, range 52-69 | 0/16 | Breast | 16 | - | All thoracic or lumbar metastases | - |
| Dabravolski et al 2015 | 65.5, range 31-92 | 94/156 | Breast | 48 | 812 | Cervical (39), thoracic (462), lumbar (311) | Radiotherapy/ chemotherapy (229) |
|  |  |  | Plasmacytoma | 39 |  |  |  |
|  |  |  | Lung | 32 |  |  |  |
|  |  |  | Renal | 26 |  |  |  |
|  |  |  | Uterine/ovarian | 25 |  |  |  |
|  |  |  | Thyroid | 19 |  |  |  |
|  |  |  | Bladder/prostate | 16 |  |  |  |
|  |  |  | Pancreas | 11 |  |  |  |
|  |  |  | GI tract | 9 |  |  |  |
|  |  |  | Melanoma | 4 |  |  |  |
| Georgy et al 2009 | 69.6, range 34-89 | 16/21 | Breast | 10 | 44 | Thoracic (20), lumbar (24) | - |
|  |  |  | Lung, myeloma | 6 |  |  |  |
|  |  |  | Plasmacytoma | 4 |  |  |  |
|  |  |  | Colon | 3 |  |  |  |
|  |  |  | Lymphoma, urethral, renal | 2 |  |  |  |
|  |  |  | Urothelial, stomach, prostate, cervix, liver | 1 |  |  |  |
| Nakatsuka et al 2009 | 61.0 (13), range 52-78 | 6/4 | Colorectal | 4 | 10 | Thoracic (3), lumbar (6), sacral (1) | Chemotherapy (9), radiotherapy (5), arterial embolization (1) |
|  |  |  | Liver | 2 |  |  |  |
|  |  |  | Oesophageal, renal, prostate, lung | 1 |  |  |  |

*data from the largest institution only, no data available for the whole study population

**Table S6. GRADE approach for meta-analysed outcomes:**

| **Study design** | **No. of Studies/ patients** | **Estimate of effect (95% CI)** | **Study Limitations** | **Imprecision** | **Inconsistency** | **Indirectness** | **Overall strength of evidence** |
| --- | --- | --- | --- | --- | --- | --- | --- |
| ***Pain (3-5 weeks follow up)*** | | | | | | | |
| Observational | 8/286 | 2.24 (1.55, 2.93) | Serious limitations* | Moderate | Moderate inconsistency** | No serious indirectness | Low |
| ***Pain (3-4 month follow up)*** | | | | | | | |
| Observational | 4/98 | 3.00 (1.11, 4.90) | Serious limitations* | Serious | Moderate inconsistency** | No serious indirectness | Very Low |
| ***Pain (5-6 month follow up)*** | | | | | | | |
| Observational | 4/144 | 3.54 (1.96, 5.11) | Serious limitations* | Serious | Moderate inconsistency** | No serious indirectness | Very low |

*serious limitations due to the serious risk of bias in the majority of studies

** Thought there was important statistical heterogeneity, the direction of effect were similar for all studies hence why inconsistency was judged as moderate and not low.

**Table S7. Data extraction table for pain, disability and HRQOL:**

| Study | Intervention(s) | Pain scale used | *n* (pre-op) | Pre-operative mean pain /baseline score (SD) | *n* (post-op) | Post-operative mean pain score (SD) | P value | Disability/health related quality of life index used | Pre-operative score/ baseline (SD) | Post-operative score (SD) | P value | Time point both outcomes were assessed |
| --- | --- | --- | --- | --- | --- | --- | --- | --- | --- | --- | --- | --- |
| Bagla et al 2016 | RFA + vertebral augmentation | NPRS | 50 | 5.9 | 49 | 3.7 | <0.0001 | MODI | 52.9 | - | - | Discharge |
|  |  |  |  |  | 45 | 3.6 | <0.0001 |  |  | 45.2 | <0.01 | Day 3 |
|  |  |  |  |  | 46 | 3.5 | <0.0001 |  |  | 45.9 | <0.01 | 1 week |
|  |  |  |  |  | 40 | 2.6 | <0.0001 |  |  | 40.0 | <0.01 | 1 month |
|  |  |  |  |  | 34 | 2.1 | <0.0001 |  |  | 37.0 | <0.01 | 3 months |
| Sayed et al 2019 | RFA + vertebral augmentation | NRS-11 | 30 | 5.77 (2.81) | 23 | 4.65 (2.82) | 0.1571 | FACT-G7 | 13.0 (3.64) | 14.7 (4.49) | 0.1341 | 3 days |
|  |  |  |  |  | 26 | 3.33 (2.59) | 0.0014 |  |  | 14.69 (4.92) | 0.1464 | 1 week |
|  |  |  |  |  | 25 | 2.64 (2.41) | 0.0001 |  |  | 14.04 (4.49) | 0.347 | 1 month |
|  |  |  |  |  | 18 | 2.61 (2.28) | 0.0002 |  |  | 15.11 (3.97) | 0.0711 | 3 months |
| Prezzano et al 2019 | RFA + vertebral augmentation  vs  RT + RFA + vertebral augmentation  *this is the mean VAS score for both interventions combined. There was no significant difference in VAS scores between RFA alone vs RFA + RT (p=0.96) | VAS | 26 | 4.52 (3.07) | - | 2.7* (2.9) | <0.0001 | - | - | - | - | 3 weeks |
|  |  |  |  |  |  | 1.6*  (2.6) | <0.0001 |  |  |  |  | 12 weeks |
| Zhao et al 2018 | RFA + vertebral augmentation | VAS | 16 | 8.1 (1.4) | 16 | 5.5 (1.1) | <0.05 | EORTC QLQ-C30 | - | - | - | 24 hours |
|  |  |  |  |  | 16 | 5.1 (1.0) | <0.05 |  | - | - | - | 48 hours |
|  |  |  |  |  | 16 | 4.8 (0.9) | <0.05 |  | - | - | - | 72 hours |
|  |  |  |  |  | 16 | 2.8 (0.6) | <0.05 |  | - | - | - | 1 week |
|  |  |  |  |  | 16 | 2.4 (0.6) | <0.05 |  | Physical function- 15.57 (1.94)  Role function -6.50 (0.91)  Emotional function- 12.89 (1.88)  Cognitive function -4.55 (1.93)  Social function-  4.54 (1.93) | Physical function-  13.48 (2.17)  Role function-  6.14(1.39)  Emotional function-  10.79(1.57)  Cognitive function-  5.22 (1.68)  Social function-  5.23 (1.68) | 0.030, 0.472, 0.003, 0.373, 0.443 | 1 month |
|  |  |  |  |  | 16 | 2.2 (0.6) | <0.05 |  | - | - | - | 2 months |
|  |  |  |  |  | 16 | 1.9 (0.8) | <0.05 |  | - | - | - | 3 months |
|  |  |  |  |  | 16 | 1.4 (0.8) | <0.05 |  | - | - | - | 6 months |
| Cazzato et al 2018 | RFA + vertebral augmentation | VAS | 11 | 7.8 (1.1) | 11 | 3.5 (2.0) | <0.01 | - | - | - | - | Median of 1 month |
| Greenwood et al 2015 | RFA + RT +vertebral augmentation | NRS | 21 | 8.0 (2.3) | 21 | 4.3 (3.1) | <0.02 | - | - | - | - | 1 week |
|  |  |  |  |  | 19 | 2.9 (3.3) | <0.0003 | - | - | - | - | 1 month |
| Anchala et al 2014 | RFA + vertebral augmentation | VAS | 92 | 7.51 (2.46) | 56 | 1.73 (2.28) | <0.0001 | - | - | - | - | 1 week |
|  |  |  |  |  | 83 | 2.25 (2.44) | <0.0001 | - | - | - | - | 1 month |
|  |  |  |  |  | 9 | 1.75 (2.62) | 0.009 | - | - | - | - | 6 months |
| Gervagez et al 2008 | RFA + vertebral augmentation | VAS | 41 | - | 26 | -  (36.2% decrease from baseline) | 0.001 | Pain disability index (PDI) | - | -  (8% decrease from baseline, *n=24*) | 0.015 | 6 weeks |
|  |  |  |  |  | 19 | -  (50% decrease from baseline) | 0.001 |  |  | -  (4% decrease from baseline, *n=21*) | 0.002 | 6 months |
|  |  |  |  |  | 14 | -  (50% decrease from baseline) | 0.002 |  |  | -  (10% decrease from baseline, *n=15*) | 0.003 | >6 months |
| Wallace et al 2015 | RFA + vertebral augmentation | NRS | 64 | 8.0 (1.9) | 64 | 3.9 (3.0) | <0.0001 | - | - | - | - | 1 week |
|  |  |  |  |  | 58 | 2.9 (3.0) | <0.0001 | - | - | - | - | 4 weeks |
| Zheng et al 2014 | RFA +vertebral augmentation | VAS | 26 | 7.69 (1.12) | 26 | 6.62 (1.02) | <0.01 | - | - | - | - | 3 days |
|  |  |  |  |  | 26 | 4.65 (1.55) | <0.01 | - | - | - | - | 1 week |
|  |  |  |  |  | 26 | 3.62 (0.98) | <0.01 | - | - | - | - | 1 month |
|  |  |  |  |  | 26 | 2.77 (0.82) | <0.01 | - | - | - | - | 3 months |
|  |  |  |  |  | 26 | 2.96 (0.92) | <0.01 | - | - | - | - | 6 months |
| Proschek et al 2009* | RFA alone | VAS | 8 | 7.9 | 8 | 5.5 | 0.018 | QoL ODI | 64% | 34% | 0.014 | Post-treatment |
|  |  |  |  |  | 8 | 4.0 | 0.008 |  |  | 33% | 0.06 | 3-6 months |
|  | RFA + vertebral augmentation | VAS | 8 | 7.6 | 8 | 5.0 | 0.005 | QoL ODI | 66% | 36% | 0.003 | Post-treatment |
|  |  |  |  |  | 8 | 3.5 | 0.005 |  |  | 35% | 0.071 | 15-36 months |
| Dabravolski et al 2015 | RFA + vertebral augmentation | VAS | 250 | 7-10 | - | 0-3 | - | - | - | - | - | Up to 60 months |
| Georgy et al 2009 | RFA + vertebral augmentation | VAS | 28 | - | 28 | 25/28 (89.5%) reported pain relief | - | - | - | - | - | 2-4 weeks |
| Nakatsuka et al 2009 | RFA + vertebral augmentation | VAS | 10 | 7.5 (2.7) | 10 | 2.7 (2.0) | 0.00005 | - | - | - | - | Mean follow up of 4.5 months |

**Table S8. Data extraction table for complications, tumour control and mortality:**

| Study | Intervention(s) | Procedural complications (*n*) | Progression/recurrence of spinal metastases | Mortality |
| --- | --- | --- | --- | --- |
| Bagla et al 2016 | RFA + vertebral augmentation | None | - | 5 patients died within 3 month follow up period (deemed unrelated to procedure) |
| Sayed et al 2019 | RFA + vertebral augmentation | None | Follow-up imaging was done for 2 patients and both showed local tumour control (followed up at 3 months) | - |
| Prezzano et al 2019 | RFA + vertebral augmentation  vs  RFA + RT + vertebral augmentation | - | Local failure in 8/17 tumours for RFA alone compared to 1/11 in RFA + RT group at median follow up of 8.2 months. | Median survival was 31.9 weeks in RFA alone vs 55.3 weeks in RFA +RT |
| Tomasian et al 2018 | RFA + vertebral augmentation) | None | Only 1/23 reported local tumour control failure (median follow up 16 weeks). Tumour control in 25/26 tumours. | 8 patients eventually died (due to other causes) and 2 patients entered hospice care due to progression of metastatic disease. |
| Zhao et al 2018 | RFA + vertebral augmentation | Cement leakage (1) causing moderate pain, needed surgical removal. | - | All patients survived (mean follow up 8.4 months) |
| Cazzato et al 2018 | RFA + vertebral augmentation) | Asymptomatic ement leakage (1), sepsis leading to death (1) | For 6/11 patients, follow up-imaging was done. 4/6 had stable control, 2/6 had progressed. (median follow up of 2.5 months) | 1 died due to sepsis 3 weeks post-procedure |
| Greenwood et al 2015 | RFA + RT + vertebral augmentation | New radicular pain (1) | 13/21 had follow- up imaging (median follow up of 6 months). At 3 months, 1/13 had tumour progression at 3 months and 0/10 at 6 months. | 1 died within 4 weeks |
| Anchala et al 2014 | RFA + vertebral augmentation | Asymptomatic cement leakage (2) | 3/13 had tumour progression (mean follow up of 82 days) | - |
| Gervagez et al 2008 | RFA + vertebral augmentation | Increasing pain in lower limbs (2), unilateral monoradiculopathy (1), thermally induced paraplegia (1).  All side effects were temporary | 33/41 had follow up imaging. 5/33 had tumour progression (mean time of 730 ±54 days to progression) | - |
| Wallace et al 2015 | RFA +vertebral augmentation | Radicular pain (4) | - | 6 died within 4 weeks. |
| Zheng et al 2014 | RFA + vertebral augmentation | None | Tumour recurrence reported in 0/26 patients (mean follow up of 8.4 ±2.1 months) | 0 deaths within follow up period (mean 8.4 months) |
| Proschek et al 2009 | RFA alone  vs  RFA + vertebral augmentation | None | Tumour recurrence reported in 0/16 patients (mean follow up of 20.4 months) |  |
| Dabravolski et al 2015 | RFA + vertebral augmentation | Cement leak (38) of no clinical relevance | Recurrence occurred in 30/229 patients recurred within 5 years | 151/229 (65.9%) survived after 1 year, 94/229 (41.1%) after 2 years, 53/229 (23.1%) after 3 years, 37/229 (16.2%) after 4 years and 34/229 (14.9%) after 5 years |
| Georgy et al 2009 | RFA + vertebral augmentation | Clinically insignificant cement (28) extravasation and epidural extravasations (2). radicular pain (1) | - | - |
| Nakatsuka et al 2009 | RFA + vertebral augmentation | Transient neural damage (1) due to high spinal canal temperature. | - | All (10) patients died within mean follow up of 4.5 months (range 2.7-7.1 months |
